# Association of Chronic Pancreatitis and Malignant Main Duct IPMN: A Rare but Difficult Clinical Problem 

**DOI:** 10.1155/2017/8705195

**Published:** 2017-02-22

**Authors:** Zoltán Berger, Hernán De La Fuente, Manuel Meneses, Fernanda Matamala, Makarena Sepúlveda, Claudia Rojas

**Affiliations:** ^1^Departamento Medicina, Sección Gastroenterología, Hospital Clínico Universidad de Chile, Santiago, Chile; ^2^Clínica Santa María, Santiago, Chile; ^3^Fundación Arturo López Pérez, Santiago, Chile; ^4^Universidad Andres Bello, Santiago, Chile; ^5^Universidad de los Andes, Santiago, Chile

## Abstract

We report the case of a 70-year-old woman who consulted for recurrent short episodes of mild-to-moderate abdominal pain. Dilated main pancreatic duct was seen on CAT scan and magnetic resonance, with multiple calcifications and intraductal stones, typical in CP. However, for a more pronounced cystic dilatation in the pancreatic head, we could not exclude the coexistence of a main duct IPMN. ERCP was performed, with pancreatic sphincterotomy and extraction of pancreatic stones, but, at the same time, mucin extrusion was seen from the dilated duct through the papilla. Pancreatoduodenectomy was performed. Surgery and histology confirmed malignant IPMN with the typical image of chronic pancreatitis and intraductal stones in the vicinity. The patient is doing well 4 years after the surgery, without recurrence of the malignant disease, with changes of chronic pancreatitis in the pancreatic remnant. This paper discusses the possible relationships between the two entities and emphasizes the need of differential diagnosis.

## 1. Introduction

Chronic pancreatitis is a chronic inflammatory disease of the pancreas, frequently with a benign clinical course. The diagnosis of the advanced forms is generally easy thanks to characteristic morphologic findings: irregularly dilated main pancreatic duct with intraductal stones, calcifications, and frequent dilation of branch ducts [1]. CP can also be complicated by pseudocysts, sometimes communicated to the main duct.

In contrast, intraductal papillary mucinous neoplasia (IPMN) is a mucin-producing tumor of ductal origin, with variable probability of malignant transformation. In the case of main duct IPMN, high-grade dysplasia and even invasive cancer are already present in 50–70% of lesions when diagnosed [2, 3] and this frequency is increasing. Differential diagnosis is based on CAT scan and magnetic resonance images (MRI): presence of pancreatic stones, diffuse dilatation of the main duct with “chain of lakes” images, and dilated branch ducts argue in favor of CP, while more circumscribed and larger cystic lesions, particularly with mural nodules, are frequent in the main duct IPMN. The therapy of the two diseases is quite different: conservative treatment or minimally invasive endoscopic procedures in CP and radical surgical resolution in main duct IPMN, with a relatively good prognosis of about 70% survival at 5 years [2].

However, differential diagnosis can be difficult, particularly if calcifications are present in the dilated duct.

We report the case of our patient, in whom CP and main duct IPMN were simultaneously detected and treated successfully by surgery.

## 2. Case Report

**Figure 1:**
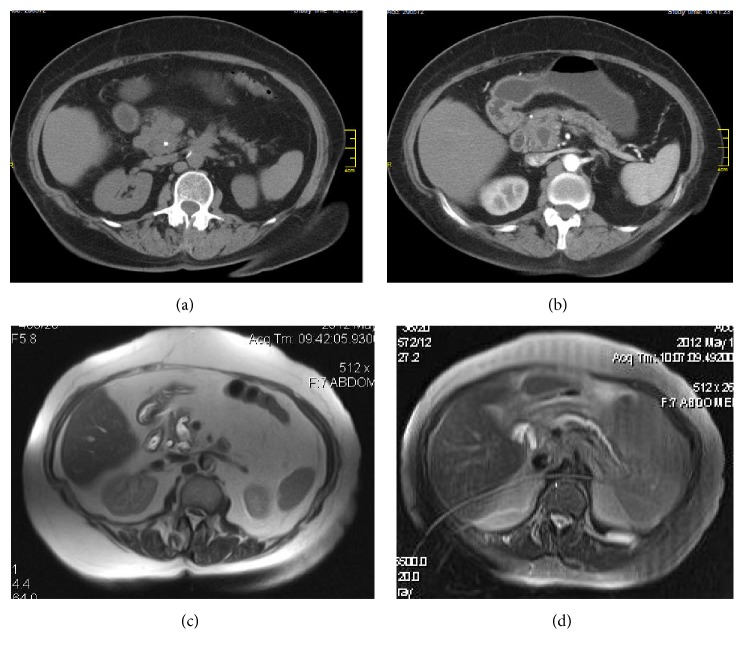
CAT scan ((a) and (b)) and magnetic resonance ((c) and (d)) images before the surgery. (a) Pancreatic head increased in size, with focal calcification, without mass lesion. (b) Dilated pancreatic duct in the whole pancreas, with major diameter, and fusiform dilatation in the pancreatic head. Slight atrophy of pancreatic parenchyma. (c) Fusiform dilated main pancreatic duct in the pancreatic head, with small filling defect suspected. (d) Dilated main pancreatic duct in pancreatic body and tail, with slight atrophy of parenchyma.

**Figure 2:**
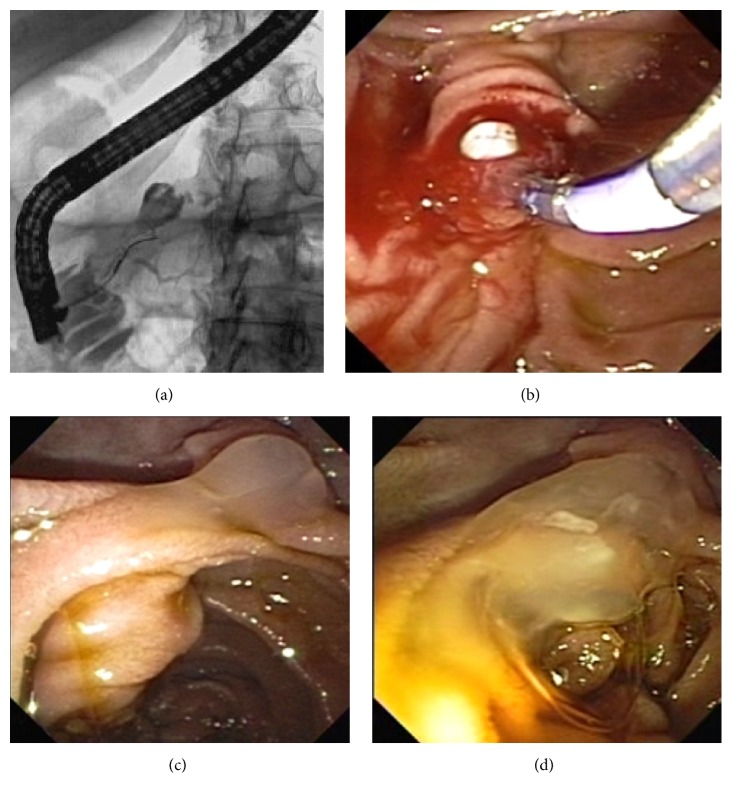
ERCP. (a) ERCP: incomplete contrast injection in the pancreatic duct. Cystic dilatation of the main pancreatic duct in the head of pancreas; initial opacification of less dilated duct in the body. Note normal caliber duct in uncinated process. (b) Pancreatic papillotomy, followed by passage of white pancreatic stone. No mucin was seen at this moment. (c) “Fish eye” sign; mucin spurring from the minor papilla. (d) Flow of mucin from the minor papilla.

**Figure 3:**
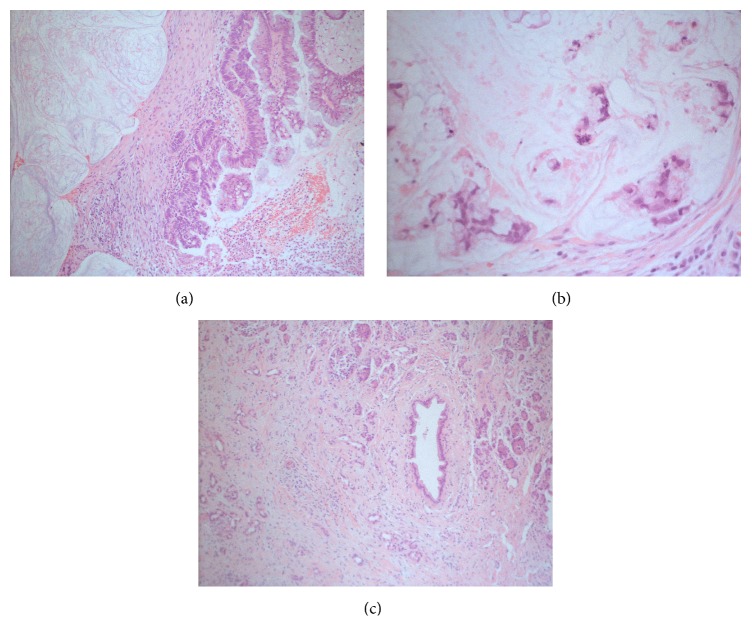
Histologic findings. Hematoxylin-eosin, 100x. (a) Neoplastic epithelium of a pancreatic duct, with formation of papillary structures (on the right). Abundant mucin formation on the left side. (b) Mucinous uniform cells with epithelial atypia and invasion of the neighborhood. (c) Tumor-free pancreatic tissue. Distortion of the normal architecture; moderate-to-severe fibrosis is seen with inflammatory cell infiltration. We can observe dilated ductal elements and atrophy of acinar cells.

A 70-year-old female patient consulted medical doctor for recurrent moderate abdominal pain in the last few months. She had had a cholecystectomy 30 years ago; later on, without significant illness in medical history, she was a teetotaler and she smoked 2 cigarettes per day during 10 years but stopped completely during the 6 last months. Routine blood tests were in the normal range. Abdominal ultrasound did not visualize the pancreas well due to bloating. A CAT scan was performed, finding multiple calcifications and irregularly dilated main pancreatic duct, predominantly in the pancreatic head (Figures 1(a) and 1(b)), where the dilatation had some cystic aspect and was disproportionally more pronounced than in the pancreatic body and tail. The same alterations were confirmed by MRI (Figures 1(c) and 1(d)), without intramural nodules in the dilated main duct. Based on the two imaging methods, we could not distinguish between chronic calcifying pancreatitis and mucin-producing neoplasia, that is, main duct IPMN. ERCP was performed, and contrast injection revealed a cystic dilation of main pancreatic duct in the head (Figure 2(a)). The duct of Wirsung was less dilated in the body. Through pancreatic papillotomy, typical whitish pancreatic stone passed into the duodenal lumen (Figure 2(b)). However, after having continued the contrast injection, mucin passage was seen producing the typical fish eye appearance in the major and also the minor papilla (Figures 2(c) and 2(d)). Simultaneous existence of CP with pancreatic stones and main duct IPMN was our final diagnosis. Given the high probability of malignancy in the dominant cystic lesion, surgery was decided and performed. Surgery revealed a cystic tumor that was found in a fibrotic pancreas. Pancreatoduodenectomy was performed with preservation of pylorus and pancreaticogastrostomy. Some whitish pancreatic stones were extracted. No lymph node metastasis was seen. Histological (Figure 3) findings confirmed the existence of a mucinous tumor emerging from the main pancreatic duct, with multiple foci of high grade dysplasia and even with evident transformation into an infiltrative malignant lesion (Figure 3). No lymph node metastasis or vascular involvement was found. Fibrosis, acinar cell degeneration, and tubular complexes [4] were seen in the surrounding tumor-free pancreatic tissue. The patient is still doing well 4 years after the intervention, without suspicion of any tumor recurrence.

## 3. Discussion

IPMN is recognized with increasing frequency, thanks to the general accessibility of imaging methods, as an incidental finding in the vast majority of cases. These mucinous benign tumors generally originate in the branch ducts. Main duct IPMN is far less frequent but a much more severe entity for its high probability of malignant transformation. Furthermore, in some cases, it is difficult to distinguish between CP and IPMN and the two entities can coexist in the same pancreas [5–9].

The presence of histological alterations of focal chronic pancreatitis near to any pancreatic tumor is a usual finding, but calcification is only exceptionally present. If calcification is seen, one can hypothesize 3 possibilities. (1) The first hypothesis is calcification of the tumor, frequent as a central scare in benign serous cystadenomas and peripheral in malignant mucinous cystadenoma. However, in these cases, the calcification is present in the tumor and not in the focal chronic pancreatitis. (2) The second hypothesis is calcification, where intraductal stones can be part of a preexistent chronic pancreatitis and the main duct IPMN can develop in a previously ill pancreas. In effect, the risk of pancreatic adenocarcinoma is higher in CP, but it is a ductal adenocarcinoma and not an IPMN with malignant transformation. (3) The third hypothesis is that IPMN, like all pancreatic tumors, can produce a focal CP, probably by partial obstruction. However, obstructive CP generally does not present stones. Zapiach et al. [5] analyzed possible mechanisms of calcification seen in their 10 patients and they found this last mechanism as a possible explanation: long-lasting partial ductal obstruction could be the cause of the stone formation in this special form of human obstructive pancreatitis. Our case seems to be in line with this hypothesis: the medical history of our patient was relatively short; some months, she had no symptoms of chronic pancreatitis earlier and she had none of the known etiological factors of the disease. Probably the slowly growing main duct IPMN was the cause of her obstructive pancreatitis with subsequent stone formation.

Our case, while not unique, seems to be rare, given the simultaneous presence of radiological signs of CP with intraductal stones and a malignant IPMN. Endoscopic therapy, extraction of stones, could have been a good therapeutic option for the recurrent pain produced by CP. On the other hand, the only curative possibility for a main duct IPMN is radical surgery [10]. Although malignant, as in our case, the prognosis is quite different from ductal adenocarcinoma, with >70% of 5-year survival. In our case, ERCP findings were also equivocal, even including pancreatic stone extraction, but finally the mucin extrusion from the papillary orifice after manipulation in the pancreatic duct permitted the final preoperative diagnosis and the correct choice of surgical therapy.

Our case emphasizes again the possibility that IMPN and chronic pancreatitis may coexist in the same patient. Morphologic signs of CP, even the presence of intraductal stones, are not enough to exclude the simultaneous presence of main duct IPMN. If radiological findings are not clear enough [11] and the suspicion of a focal lesion persists, every possible effort must be made to reach a precise diagnosis, since the distinction between these two entities is of vital importance for the patient. In our case, we considered a high probability of CP without IPMN on the basis of CT and MRI. ERCP was thus chosen as first procedure with diagnostic and therapeutic purposes. However, EUS and fine-needle biopsy could be also the first, less invasive step. The finding of mucin defined the nature of the lesion without any other procedure, but in some cases EUS, fine-needle biopsy, and ERCP could be necessary to establish the final diagnosis.
